# Timing of Exercise Affects Glycemic Control in Type 2 Diabetes Patients Treated with Metformin

**DOI:** 10.1155/2018/2483273

**Published:** 2018-03-29

**Authors:** Tao Huang, Chunyan Lu, Moritz Schumann, Shenglong Le, Yifan Yang, Haihui Zhuang, Qingwei Lu, Jinsheng Liu, Petri Wiklund, Sulin Cheng

**Affiliations:** ^1^Department of Physical Education, Shanghai Jiao Tong University, Shanghai, China; ^2^Department of Endocrinology, West China Hospital, Sichuan University, Chengdu, China; ^3^Department of Molecular and Cellular Sport Medicine, German Sport University Cologne, Cologne, Germany; ^4^Faculty of Sport and Health Sciences, University of Jyväskylä, Jyväskylä, Finland; ^5^School of Life Sciences and Biotechnology, Shanghai Jiao Tong University, Shanghai, China; ^6^Jiangchuan Community Health Service Center, Shanghai, China; ^7^School Infirmary, Shanghai Jiao Tong University, Shanghai, China; ^8^The Key Laboratory of Systems Biomedicine, Ministry of Education, Shanghai Center for Systems Biomedicine, Shanghai Jiao Tong University, Shanghai, China

## Abstract

**Objective:**

The purpose of the study was to examine the acute effects of the timing of exercise on the glycemic control during and after exercise in T2D.

**Methods:**

This study included 26 T2D patients (14 women and 12 men) who were treated with metformin. All patients were tested on four occasions: metformin administration alone (Metf), high-intensity interval training (HIIT) performed at 30 minutes (EX30), 60 minutes (EX60), and 90 minutes (EX90) postbreakfast, respectively. Glucose, insulin, and superoxide dismutase (SOD) activity were examined.

**Results:**

Glucose decreased significantly after the exercise in EX30, EX60, and EX90. Compared with Metf, the decline in glucose immediately after the exercise was larger in EX30 (−2.58 mmol/L; 95% CI, −3.36 to −1.79 mmol/L; *p* < 0.001), EX60 (−2.13 mmol/L; 95% CI, −2.91 to −1.34 mmol/L; *p* < 0.001), and EX90 (−1.87 mmol/L; 95% CI, −2.65 to −1.08 mmol/L; *p* < 0.001), respectively. Compared with Metf, the decrease in insulin was larger in EX30 and EX60 (both *p* < 0.001).

**Conclusions:**

Timing of exercise is a factor to consider when prescribing exercise for T2D patients treated with metformin. This trial is registered with ChiCTR-IOR-16008469 on 13 May 2016.

## 1. Introduction

Type 2 diabetes (T2D) is considered as one of the most prevalent diseases, affecting over 250 million patients worldwide [1]. In China, the number of adults affected by T2D was estimated to be 113.9 million in 2010, while 493.4 million adults were estimated to be prediabetic [2]. T2D is generally characterized by relative insulin deficiency by pancreatic *β*-cell and impaired insulin-stimulated glucose uptake and disposal in target tissues [3]. Moreover, diabetes predisposes individuals to an increased risk of mortality induced by a range of cardiovascular and noncardiovascular diseases [4].

Metformin has been widely prescribed as an important drug for treating T2D for decades [5], and it is recommended as the first-line pharmacological therapy for newly diagnosed T2D [6, 7]. Although the mechanisms underlying the action of metformin are not fully understood, evidence suggests that metformin exerts its antidiabetic effects primarily by inhibiting hepatic glucose production as well as by increasing insulin sensitivity [8, 9]. In addition to metformin therapy, lifestyle changes (e.g., regular physical activity and hypocaloric diet) are also recommended interventions for patients with T2D [6, 7, 10].

It is well known that exercise not only increases physical fitness [11] but also leads to increased glucose uptake by skeletal muscle and stabilized blood glucose concentrations, while it chronically enhances insulin sensitivity and decreases concentrations of glycosylated hemoglobin (HbA_1c_) [12–15]. However, the optimal exercise prescription for T2D has not been established [14]. Moreover, the combined effects of exercise and metformin therapy on glycemic control have not been well documented and the current literature yielded conflicting results. Specifically, studies demonstrated that concurrent exercise and metformin administration may blunt the acute effects of a single exercise session on insulin sensitivity [16] and attenuate the chronic effects of exercise training on insulin sensitivity and some risk factors of cardiovascular diseases [17, 18]. Conversely, another study reported that metformin did not attenuate the benefits of exercise training on glycemic control and fitness [19]. Furthermore, two recent studies showed that postbreakfast exercise had a positive effect on glycemic control in patients treated with metformin [20, 21].

However, the timing of exercise was not considered in previous studies. It remains unclear whether the effects on glycemic control are affected by the timing of exercise in T2D patients treated with metformin. Therefore, the purpose of the present study was to investigate the effects of exercise timing on the glycemic control during and after exercise in T2D.

## 2. Materials and Methods

### 2.1. Participants

Participants were recruited from two clinical health-care centers in China using a two-step screening procedure. Firstly, approximately 2523 patients with T2D were screened from the local diabetes database and clinical outpatient registration. The screening criterion at this stage was diagnosed type 2 diabetes within 5 years. A nurse contacted these potential participants by phone to ascertain the use of medication and complications. Out of the 100 potential participants who met the inclusion criteria, 56 were interested and invited to a laboratory visit. During this visit, detailed information regarding the study was provided and the potential participants completed a screening and health questionnaire. The eligibility criteria for this study were as follows: men and women (30–65 years old) diagnosed with T2D no more than 5 years and prescribed with metformin (maximal daily dose of 2000 mg). Exclusion criteria included the following: diagnosed comorbidities such as cardiovascular diseases, musculoskeletal diseases, or mental diseases; body mass index (BMI) greater than 38 kg/m^2^; or diagnosed type 1 diabetes mellitus. Finally, 34 patients participated in the study. Of those, 26 patients (14 women and 12 men, mean age = 53.8 ± 8.6 years) completed all testing procedure and were included in this report. All patients were informed about possible risks of all study procedures prior to testing. The study was conducted according to the Declaration of Helsinki. Ethics approval was obtained from the Ethics Committee of Bio-X center at Shanghai Jiao Tong University (number ML16027) and West China Hospital at Sichuan University (number 2016189). Written informed consent was obtained from each participant prior to enrolment in the study.

### 2.2. Study Design

**Figure 1:**
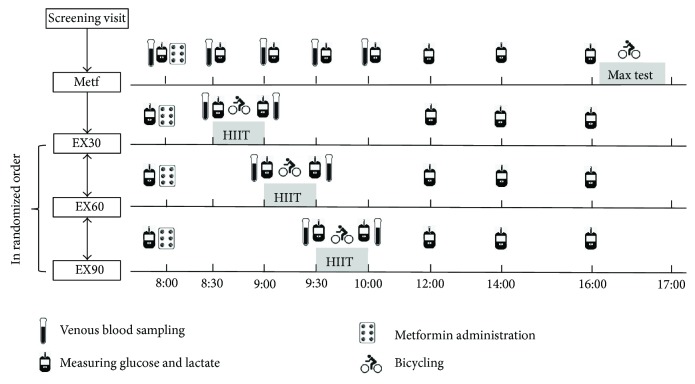
Chart of study design. Each patient underwent all of the four experimental regimens: (1) Metf: metformin administration alone; (2) EX30: HIIT performed at 30 minutes postbreakfast; (3) EX60: HIIT performed at 60 minutes postbreakfast; (4) EX90: HIIT performed at 90 minutes postbreakfast.

This study was a randomized crossover trial. Subjects reported to the laboratory on four different occasions, each separated by a minimum of one day (Figure 1). Therefore, each exercise session was separated by a minimum of 48 hours. During the first visit, subjects' diurnal glucose metabolism was assessed between 8:00 a.m. and 4:00 p.m. (Metf). Thereafter, subjects were familiarized with the cycle ergometer and tested for maximal aerobic power by an incremental cycle ergometer test. During the subsequent laboratory visits, subjects performed a single session of high-intensity interval training (HIIT) 30 (EX30), 60 (EX60), or 90 (EX90) minutes following breakfast and metformin administration in a randomized order. The three exercising time points were selected within the mid-postprandial period (30–90 min postmeal), which was previously recommended as time window for diabetes patients to manage hyperglycemia through moderate-intensity exercise, with minimal risk of hypoglycemia [22].

Subjects arrived in a fasted state on each laboratory visit. Standardized breakfast and metformin were administered at 8:00 a.m. On exercise days, cycling was performed at 8:30 a.m. (EX30), 9:00 a.m. (EX60), or 9:30 a.m. (EX90), respectively. Capillary blood was collected from the fingertip for assessing blood glucose and lactate concentrations. Venous blood samples were drawn before and immediately after the cycling exercise.

### 2.3. Metformin Administration

Metformin was prescribed by the participants' doctors. The participants did not change their medication during the study. The prescribed daily dose of metformin ranged from 500 mg to 1700 mg.

### 2.4. Anthropometrics, Blood Pressure (BP), and Physical Activity

Body height and weight were measured using standardized procedures. Resting BP was measured by an electronic BP monitor (HEM-7051, Omron Healthcare Co. Ltd., Dalian, China). The Chinese version of the International Physical Activity Questionnaire (IPAQ) short form was used to obtain daily physical activity level [23].

### 2.5. Meal Standardization

Dietary intake was standardized on the evening prior to each laboratory day as well as between 8:00 a.m. and 4:00 p.m. during each testing day. The meals were provided by the research personnel and eaten at the laboratory. The timing of meals was as follows: dinner 6:30 p.m., breakfast 8:00 a.m., lunch 12:00 p.m., snacks one hour after exercise cessation and 3:00 p.m. The lunch accounted for 40% of the total daily energy intake. The breakfast and dinner each accounted for 30% of the total daily energy intake, respectively. The total energy of meals ranged from approximately 1400 to 1800 kcal, which was adjusted individually based on body weight. The proportion of macronutrients were as follows: 40–50% carbohydrate (fiber > 20 g), 20–30% fat (saturated fatty acids 10%, mono-unsaturated fatty acids 15–20%, and poly-unsaturated fatty acids 10%), and 20% protein, respectively. Water was allowed throughout all testing days ad libitum.

### 2.6. Maximal Aerobic Capacity

All exercise tests were supervised by a clinical doctor. ECG (Zephyr Wireless Monitor, Zephyr Technology Corp., New Zealand) and oxygen saturation (Patient Monitor PM-900, Biocare, Shenzhen, China) were recorded throughout each exercise test. Subjects' maximal aerobic power was determined by an incremental cycle ergometer test (Ergomedic 839E, Monark Exercise AB, Varberg, Sweden). The initial load for all subjects was 30 watts, and the load was increased every 2 minutes by 20 watts until volitional exhaustion. Heart rate (HR) and rating of perceived exertion (RPE) were determined following each increment. Verbal encouragement was provided throughout the test. Maximal voluntary exhaustion was accepted with an RPE score ≥ 17. Maximal aerobic power (*W*_max_) was determined by the following equation [24]:

(1) $$\begin{array}{c} W_{\max}=W_{com}+\frac{t}{120}*20, \end{array}$$

where *W*_com_ is the load of the last completed stage and *t* (second) is the time of the last incomplete stage.

### 2.7. HIIT Protocol

The HIIT protocol was performed on the same cycle ergometer (Ergomedic 839E, Monark Exercise AB, Varberg, Sweden). Based on previous studies [25, 26] and pilot tests performed prior to commencing with the study, the protocol consisted of 6 1-minute bouts of high-intensity cycling at 85% of the maximal watts, separated by 3-minute bouts at 40%, leading to a total exercise duration of 27 minutes. Blood lactate and glucose concentrations were determined after each high-intensity bout as well as immediately postexercise, while RPE and HR were recorded both before and after each high-intensity bout.

### 2.8. Blood Sampling and Analysis

Venous blood samples (5 mL) were drawn from the antecubital vein in the morning after 12 hours overnight fasting on Metf day, as well as before and after the HIIT in EX30, EX60, and EX90. Serum was separated within 30 minutes and stored at −80°C until analysis. Serum glucose, total cholesterol, high-density lipoprotein cholesterol (HDL-C), low-density lipoprotein cholesterol (LDL-C), and triglycerides were analyzed by the enzymatic, colorimetric method by an automatic biochemical analyzer (Mindray BS-220, Shenzhen, China). Insulin was measured by an electrochemiluminescence immunoassay on a Cobas e411 (Roche Diagnostics International Ltd., Rotkreuz, Switzerland). HbA_1c_ was measured by chromatographic analysis on a Bio-Rad D-10 (Bio-Rad Laboratories Shanghai Ltd., Shanghai, China). Total superoxide dismutase (SOD) activity was analyzed using the spectrophotometric method on a Modular P800 analyzer (Roche Diagnostics International AG, Rotkreuz, Switzerland). Capillary blood samples were collected from the fingertip at 8:00 a.m., 8:30 a.m., 9:00 a.m., 9:30 a.m., 10:00 a.m., 12:00 p.m., 2:00 p.m., and 4:00 p.m. in Metf and at 8:00 a.m., 12:00 p.m., 2:00 p.m., 4:00 p.m. in EX30, EX60, and EX90, as well as during the exercise intervention. Blood glucose concentrations were measured immediately after sampling using Omron AS1 glucose test strip and HGM-114 analyzer (Omron Healthcare Co. Ltd., Dalian, China). The coefficient of variation (CV) of the test strip was ≤5.0%. Blood lactate concentrations were assessed using ARKRAY Lactate Pro 2 test strip and analyzer (CV < 4.3%, ARKRAY Inc., Kyoto, Japan).

### 2.9. Statistics

Gender differences in the descriptive data were evaluated using an unpaired *t*-test or Wilcoxon rank sum test (nonnormally distributed data). Linear mixed-effects modelling was conducted to assess the differences between experimental conditions in the measures of glucose allowing for repeated measurements from the same individuals. Since there is a gender differences in glucose kinetics during and after exercise [27], the potential interaction between gender and experimental conditions was tested by adding a gender × experimental interaction term in the model. No significant interactions were found. Therefore, the interaction term was removed from the final model. Moreover, considering that the doses of metformin may modify the timing effects of medication and exercise, a dose × experimental interaction term was then added in the model. Since no significant modification effects of doses were observed, the interaction term was removed from the final model. All statistical analyses were conducted with STATA 14 for Windows (StataCorp, College Station, Texas, USA), and the level of significance was set at *p* < 0.05 (two-sided).

## 3. Results

### 3.1. Participant Characteristics

Out of the 34 participants, 26 completed all test procedures. Four patients dropped out due to personal reasons (i.e., not available on testing days). Four patients were excluded before exercise tests due to unreported diseases or medication. The analyses were conducted on the 26 participants. Physical characteristics, anthropometrics, biochemical information, and metformin dose in the morning are presented in Table 1. Men were heavier and taller than women (both *p* < 0.05). Men also had greater maximal aerobic capacity than women (*p* < 0.05). No significant differences of other baseline characteristics were observed between women and men.

### 3.2. Changes in Glucose during Exercise

**Figure 2:**
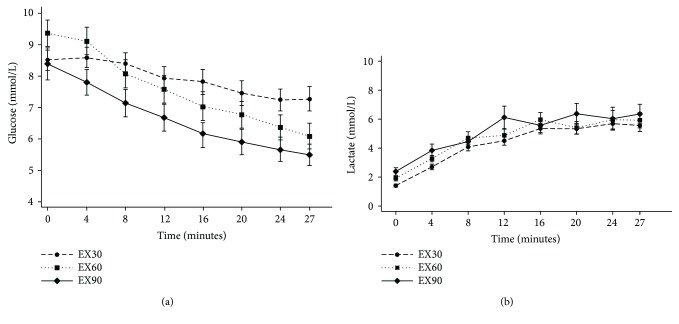
Glucose (a) and lactate (b) concentrations during the three exercise sessions. Long dash: EX30; short dash: EX60; solid line: EX90. Data are expressed as mean (SEM).

Capillary glucose decreased significantly after exercise in EX30, EX60, and EX90 (Figure 2(a) and Supplementary Table 1). Compared with EX30, reductions in glucose concentrations were significantly larger after exercises in EX60 (−2.03 mmol/L; 95% CI, −2.67 to −1.39 mmol/L; *p* < 0.001) and EX90 (−1.66 mmol/L; 95% CI, −2.30 to −1.02 mmol/L; *p* < 0.001). Blood lactate concentrations were significantly increased to a similar extent during all three exercise sessions (Figure 2(b)).

### 3.3. Glucose during the Day

**Figure 3:**
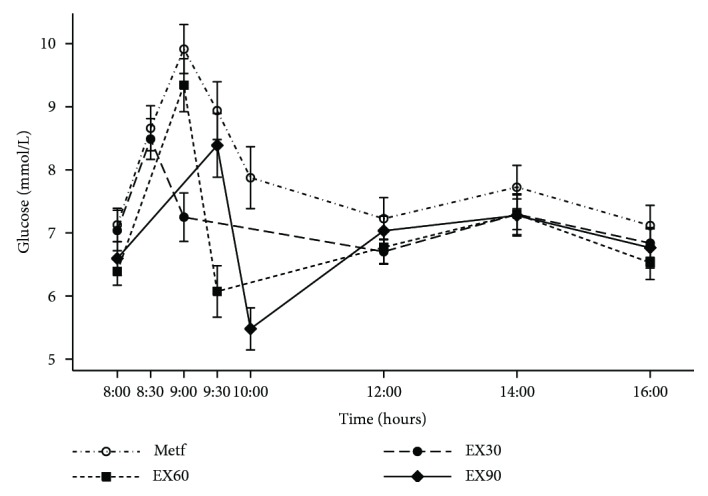
Changes of blood glucose levels throughout the four experimental conditions. Short dash and dot: Metf; long dash: EX30; short dash: EX60; solid line: EX90. Data are expressed as mean (SEM).

Changes in capillary blood glucose during the day are presented in Figure 3 and Supplementary Table 2. On the Metf day, the postprandial peak glucose was observed after one hour and began to decrease thereafter. Compared with Metf, the declines in glucose immediately after exercise (postexercise versus fasting) were larger in EX30 (−2.58 mmol/L; 95% CI, −3.36 to −1.79 mmol/L; *p* < 0.001), EX60 (−2.13; 95% CI, −2.91 to −1.34; *p* < 0.001), and EX90 (−1.87 mmol/L; 95% CI, −2.65 to −1.08 mmol/L; *p* < 0.001). The declines in EX30 day were larger than that in EX90 day (*p* = 0.04). There are no significant differences when comparing EX30 day with EX60 day and comparing EX60 day with EX90 day. The changes in glucose in the remaining hours after the exercise (i.e., at 12:00 p.m., 2:00 p.m., and 4:00 p.m.) did not differ between the four experimental conditions (all *p* > 0.05), although there was a tendency that exercising days had lower values compared with Metf.

### 3.4. Insulin and SOD Activity

**Figure 4:**
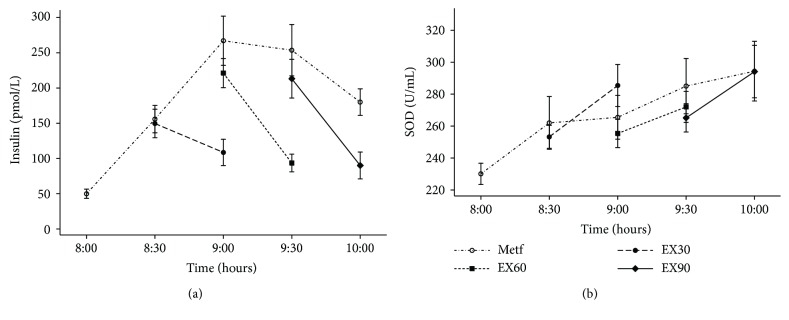
Changes of insulin (a) and SOD (b) in the four experimental conditions. Short dash and dot: Metf; long dash: EX30; short dash: EX60; solid line: EX90. Data are expressed as mean (SEM).

The pattern of changes in insulin was similar to the changes in glucose. Compared with Metf, decreases in insulin (postexercise versus preexercise) were larger in EX30 (−152.14 pmol/L; 95% CI, −215.70 to −88.57 pmol/L; *p* < 0.001) and EX60 (−110.41 pmol/L; 95% CI, −173.94 to −46.87; *p* < 0.001) (Figure 4(a)). In EX90, the decrease in insulin did not significantly differ from that in Metf (−46.35 pmol/L; 95% CI, −110.86 to 18.16 pmol/L; *p* = 0.16). Total SOD activity was increased from 8:00 a.m. to 10:00 p.m. in Metf; however, the changes in SOD in EX30, EX60, and EX90 did not significantly differ from Metf (Figure 4(b)).

## 4. Discussion

The current study examined the acute effects of the timing of exercise on the glycemic control during and after exercise in T2D. The findings indicated that the timing of exercise may be a modifiable factor influencing postexercise glycemic control when combining exercise with metformin therapy.

HIIT is a time-efficient exercise mode to improve cardiovascular fitness and some cardiometabolic risk factors in patients with cardiometabolic disease [28, 29]. Recently, HIIT gained its popularity in patients with T2D [30]. In the current study, a single bout of HIIT was performed at 30 minutes, 60 minutes, and 90 minutes postbreakfast, which led to continuous decreases in blood glucose and insulin as well as increases in blood lactate concentrations during the exercise session. Our results were in accordance with previous studies [31, 32], which supported the beneficial role of HIIT in glycemic control. A recent study by Hansen et al. [20] examined the effects of metformin on glucose kinetics during a bout of 45 min moderate exercise. They showed that the combined effects of metformin and exercise improved glucose metabolic clearance rate with no risk of hypoglycemia. Therefore, the authors concluded that metformin and exercise can be administered in combination. Our findings also confirmed the findings from Erickson et al. [21] who showed that a signal bout of exercise at 30 minutes postbreakfast had a significant glucose-lowering effect in people treated with metformin monotherapy. However, the current study extended previous findings by examining the timing effects of exercise on glycemic responses to a standardized meal and metformin administration. When a bout of HIIT was performed at 30 minutes postbreakfast, the peak glucose was blunted, thereby further stabilizing the postprandial glucose fluctuation. This finding has clinical implications, since glycemic fluctuations are a therapeutic target for managing T2D [33, 34] and high glycemic fluctuations were previously linked with increased oxidative stress and a number of complications [34–36]. This exercise condition also led to larger reductions in glucose levels compared with exercise being carried out at 90 minutes postbreakfast. Although exercising at 90 minutes postbreakfast did not cause hypoglycemia, the absolute postexercise glucose concentration was the lowest among the three experimental conditions. Taken together, the results suggest that timing of exercise is a modifiable factor influencing postexercise glycemic control and exercise at 30 minutes postbreakfast may be preferred in terms of lowering and stabilizing postprandial glucose levels in patients treated with metformin.

In a previous study, Boule et al. [37] revealed that glucose response to a standardized breakfast was reduced by metformin. However, when a 35 min exercise was performed about 2.5 hours after breakfast and metformin administration, the reduction in glucose was attenuated during the 2 h postlunch period. Sharoff et al. [16] examined the combined effects of a short-term (2-3 weeks) metformin treatment and a single bout of exercise on insulin sensitivity in insulin-resistant subjects. Their results indicated that metformin might attenuate the beneficial effects of exercise alone on insulin action. Interestingly, the current results were contrary to those two studies. In the current study, the changes in glucose before lunch as well as 2 and 4 hours after lunch did not differ among the four experimental conditions. However, the discrepancy between previous studies and the current study should be interpreted with caution due to the differences in study subjects and experimental procedures.

Systemic oxidative stress is found in insulin resistance and T2D [38, 39]. Both acute and regular exercises can activate antioxidant enzymes [40]. SOD is an antioxidant which enzymatically converts superoxide into hydrogen peroxide. Considering that metformin can reduce ROS production [41], while intense exercise and muscle contraction typically lead to acute increases in ROS production [42], it was previously speculated that ROS may play a role in the combined effects of exercise and metformin on metabolic adaptation [41]. However, in the current study, postexercise changes in SOD activity did not differ from those in Metf. However, it should be noted that SOD is only one indirect marker of oxidative stress and future studies should incorporate direct indicators of ROS production, such as 8-epi-prostaglandin F2 [43], in order to obtain a clear picture on the potential interaction of exercise and metformin on glucose metabolism.

The current study had its strength and limitations. The patients of this study were only prescribed with metformin for treating T2D. This ruled out the potential interference of other glucose-lowering medication. Therefore, this population allowed us to solely investigate the combined impact of exercise with metformin therapy but at the same time, the present findings may not be applicable to patients prescribed with multiple glucose-lowering drugs. Another limitation is that no exercise only group was included in the study, which limits the potential of the study to clarify whether the combination of exercise and metformin is better than exercise alone. However, due to the considerations of ethicality and safety, we did not and cannot require the patients to withdraw their daily medication. Furthermore, the current study only investigated the effects of postbreakfast exercise and its timing. Therefore, exercising at other time points (e.g., before breakfast or surrounding the meals) may affect glycemic control differently, which needs to be considered in future studies. Meanwhile, HIIT was employed in the study. It is therefore possible that the findings may not be generalized to other forms of exercise. Finally, the insulin sensitivity was not assessed, nor were the potential long-term effects of combining exercise with metformin therapy on glycemic control evaluated. Boule et al. [19] recently showed that metformin did not attenuate the beneficial effects of a 6-month exercise training program on HbA_1c_, fasting glucose and physical fitness in T2D. However, it remains to elucidate the optimal timing of exercise in order to obtain optimal short-term and long-term benefits for T2D patients treated with metformin therapies.

## 5. Conclusions

Timing of exercise is a factor to consider when prescribing exercise for T2D patients treated with metformin. However, further studies are warranted to elucidate the long-term effects of the combination of exercise and metformin on glycemic control, as well as the underlying mechanisms.

## Figures and Tables

**Table 1:** Baseline characteristics.

Characteristics	Men (*n* = 12)	Women (*n* = 14)	All (*n* = 26)
Age (years)	53.3 ± 9.3	54.4 ± 8.3	53.8 ± 8.6
Height (cm)	167.1 ± 5.0	156.4 ± 6.8^∗^	161.4 ± 8.0
Weight (kg)	73.5 ± 8.4	61.4 ± 12.7^∗^	67.0 ± 12.4
BMI (kg/m^2^)	26.3 ± 2.9	24.9 ± 3.9	25.6 ± 3.5
Fasting glucose (mmol/L)	6.42 ± 1.13	6.42 ± 1.49	6.42 ± 1.31
Fasting insulin (pmol/L)	44.50 ± 22.60	53.29 ± 37.14	49.24 ± 31.01
HbA_1c_ (%)	6.53 ± 0.87	6.24 ± 0.68	6.37 ± 0.77
HbA_1c_ (mmol/mol)	47.80 ± 9.53	44.72 ± 7.44	46.14 ± 8.44
Total cholesterol (mmol/L)	4.19 ± 0.62	4.32 ± 0.80	4.26 ± 0.71
Triglyceride (mmol/L)^#^	1.26 (1.01, 2.07)	1.65 (0.94, 2.48)	1.35 (0.94, 2.48)
HDL-C (mmol/L)^#^	0.92 (0.70, 1.30)	1.05 (0.84, 1.50)	0.94 (0.80, 1.30)
LDL-C (mmol/L)	2.72 ± 0.59	2.52 ± 0.56	2.62 ± 0.57
Systolic BP (mmHg)	121 ± 13	131 ± 17	127 ± 16
Diastolic BP (mmHg)	73 ± 12	77 ± 9	75 ± 10
Maximal aerobic power (W)	160 ± 46	106 ± 30^∗^	131 ± 46
Physical activity (MET min/week)^#^	1386 (693, 3486)	1559 (1053, 2772)	1386 (693, 2772)
Metformin dose with breakfast (g)^##^			
0.25	4	3	7
0.5	5	7	12
0.85	3	4	7

Data are expressed as mean (SD) or median (interquartile range). BP: blood pressure; BMI: body mass index; HbA_1c_: glycosylated hemoglobin; HDL-C: high-density lipoprotein cholesterol; IPAQ: International Physical Activity Questionnaire; LDL-C: low-density lipoprotein cholesterol; MET: metabolic equivalent of task. ^#^Values expressed as median (interquartile range) due to nonnormality. ^##^Values were counts. ^∗^Significant difference between gender (*p* < 0.05).

## Data Availability

The data of the study can be obtained on request.

## Supplementary Materials

Supplementary Materials Supplementary Table 1: blood glucose concentrations during exercise in EX30, EX60, and EX90. Supplementary Table 2: blood glucose during the four experimental days.

## References

[1] Shaw J. E., Sicree R. A., Zimmet P. Z. (2010). Global estimates of the prevalence of diabetes for 2010 and 2030. *Diabetes Research and Clinical Practice*.

[2] Xu Y., Wang L., He J. (2013). Prevalence and control of diabetes in Chinese adults. *JAMA*.

[3] Chatterjee S., Khunti K., Davies M. J. (2017). Type 2 diabetes. *The Lancet*.

[4] Bragg F., Holmes M. V., Iona A. (2017). Association between diabetes and cause-specific mortality in rural and urban areas of China. *JAMA*.

[5] Foretz M., Guigas B., Bertrand L., Pollak M., Viollet B. (2014). Metformin: from mechanisms of action to therapies. *Cell Metabolism*.

[6] American Diabetes Association (2016). 7. Approaches to glycemic treatment. *Diabetes Care*.

[7] Nathan D. M., Buse J. B., Davidson M. B. (2009). Medical management of hyperglycaemia in type 2 diabetes mellitus: a consensus algorithm for the initiation and adjustment of therapy: a consensus statement from the American Diabetes Association and the European Association for the Study of Diabetes. *Diabetologia*.

[8] Hundal R. S., Krssak M., Dufour S. (2000). Mechanism by which metformin reduces glucose production in type 2 diabetes. *Diabetes*.

[9] An H., He L. (2016). Current understanding of metformin effect on the control of hyperglycemia in diabetes. *Journal of Endocrinology*.

[10] Greco M., Chiefari E., Montalcini T. (2014). Early effects of a hypocaloric, Mediterranean diet on laboratory parameters in obese individuals. *Mediators of Inflammation*.

[11] Mensink M., Blaak E. E., Wagenmakers A. J., Saris W. H. (2005). Lifestyle intervention and fatty acid metabolism in glucose-intolerant subjects. *Obesity Research*.

[12] Goodyear L. J., Kahn B. B. (1998). Exercise, glucose transport, and insulin sensitivity. *Annual Review of Medicine*.

[13] Koivisto V. A., Yki-Järvinen H., DeFronzo R. A. (1986). Physical training and insulin sensitivity. *Diabetes/Metabolism Reviews*.

[14] O’Hagan C., De Vito G., Boreham C. A. G. (2013). Exercise prescription in the treatment of type 2 diabetes mellitus: current practices, existing guidelines and future directions. *Sports Medicine*.

[15] Church T. S., Blair S. N., Cocreham S. (2010). Effects of aerobic and resistance training on hemoglobin A_1c_ levels in patients with type 2 diabetes: a randomized controlled trial. *JAMA*.

[16] Sharoff C. G., Hagobian T. A., Malin S. K. (2010). Combining short-term metformin treatment and one bout of exercise does not increase insulin action in insulin-resistant individuals. *American Journal of Physiology-Endocrinology and Metabolism*.

[17] Malin S. K., Gerber R., Chipkin S. R., Braun B. (2012). Independent and combined effects of exercise training and metformin on insulin sensitivity in individuals with prediabetes. *Diabetes Care*.

[18] Malin S. K., Nightingale J., Choi S. E., Chipkin S. R., Braun B. (2013). Metformin modifies the exercise training effects on risk factors for cardiovascular disease in impaired glucose tolerant adults. *Obesity*.

[19] Boule N. G., Kenny G. P., Larose J., Khandwala F., Kuzik N., Sigal R. J. (2013). Does metformin modify the effect on glycaemic control of aerobic exercise, resistance exercise or both?. *Diabetologia*.

[20] Hansen M., Palsoe M. K., Helge J. W., Dela F. (2015). The effect of metformin on glucose homeostasis during moderate exercise. *Diabetes Care*.

[21] Erickson M. L., Little J. P., Gay J. L., McCully K. K., Jenkins N. T. (2017). Postmeal exercise blunts postprandial glucose excursions in people on metformin monotherapy. *Journal of Applied Physiology*.

[22] Chacko E. (2017). A time for exercise: the exercise window. *Journal of Applied Physiology*.

[23] Macfarlane D. J., Lee C. C. Y., Ho E. Y. K., Chan K. L., Chan D. T. S. (2007). Reliability and validity of the Chinese version of IPAQ (short, last 7 days). *Journal of Science and Medicine in Sport*.

[24] Kuipers H., Verstappen F. T., Keizer H. A., Geurten P., van Kranenburg G. (1985). Variability of aerobic performance in the laboratory and its physiologic correlates. *International Journal of Sports Medicine*.

[25] Revdal A., Hollekim-Strand S. M., Ingul C. B. (2016). Can time efficient exercise improve cardiometabolic risk factors in type 2 diabetes? A pilot study. *J Sports Sci Med.*.

[26] Robinson E., Durrer C., Simtchouk S. (2015). Short-term high-intensity interval and moderate-intensity continuous training reduce leukocyte TLR4 in inactive adults at elevated risk of type 2 diabetes. *Journal of Applied Physiology*.

[27] Horton T. J., Grunwald G. K., Lavely J., Donahoo W. T. (2006). Glucose kinetics differ between women and men, during and after exercise. *Journal of Applied Physiology*.

[28] Weston K. S., Wisloff U., Coombes J. S. (2014). High-intensity interval training in patients with lifestyle-induced cardiometabolic disease: a systematic review and meta-analysis. *British Journal of Sports Medicine*.

[29] Batacan R. B., Duncan M. J., Dalbo V. J., Tucker P. S., Fenning A. S. (2017). Effects of high-intensity interval training on cardiometabolic health: a systematic review and meta-analysis of intervention studies. *British Journal of Sports Medicine*.

[30] Cassidy S., Thoma C., Houghton D., Trenell M. I. (2017). High-intensity interval training: a review of its impact on glucose control and cardiometabolic health. *Diabetologia*.

[31] Gillen J. B., Little J. P., Punthakee Z., Tarnopolsky M. A., Riddell M. C., Gibala M. J. (2012). Acute high-intensity interval exercise reduces the postprandial glucose response and prevalence of hyperglycaemia in patients with type 2 diabetes. *Diabetes, Obesity and Metabolism*.

[32] Karstoft K., Christensen C. S., Pedersen B. K., Solomon T. P. J. (2014). The acute effects of interval- vs continuous-walking exercise on glycemic control in subjects with type 2 diabetes: a crossover, controlled study. *The Journal of Clinical Endocrinology & Metabolism*.

[33] Frontoni S., Di Bartolo P., Avogaro A., Bosi E., Paolisso G., Ceriello A. (2013). Glucose variability: an emerging target for the treatment of diabetes mellitus. *Diabetes Research and Clinical Practice*.

[34] Monnier L., Mas E., Ginet C. (2006). Activation of oxidative stress by acute glucose fluctuations compared with sustained chronic hyperglycemia in patients with type 2 diabetes. *JAMA*.

[35] Rizzo M. R., Marfella R., Barbieri M. (2010). Relationships between daily acute glucose fluctuations and cognitive performance among aged type 2 diabetic patients. *Diabetes Care*.

[36] Torimoto K., Okada Y., Mori H., Tanaka Y. (2013). Relationship between fluctuations in glucose levels measured by continuous glucose monitoring and vascular endothelial dysfunction in type 2 diabetes mellitus. *Cardiovascular Diabetology*.

[37] Boule N. G., Robert C., Bell G. J. (2011). Metformin and exercise in type 2 diabetes: examining treatment modality interactions. *Diabetes Care*.

[38] Gopaul N. K., Anggard E. E., Mallet A. I., Betteridge D. J., Wolff S. P., Nourooz-Zadeh J. (1995). Plasma 8-epi-PGF_2*α*_ levels are elevated in individuals with non-insulin dependent diabetes mellitus. *FEBS Letters*.

[39] Meigs J. B., Larson M. G., Fox C. S., Keaney J. F., Vasan R. S., Benjamin E. J. (2007). Association of oxidative stress, insulin resistance, and diabetes risk phenotypes: the Framingham Offspring Study. *Diabetes Care*.

[40] Radak Z., Zhao Z., Koltai E., Ohno H., Atalay M. (2013). Oxygen consumption and usage during physical exercise: the balance between oxidative stress and ROS-dependent adaptive signaling. *Antioxidants & Redox Signaling*.

[41] Malin S. K., Braun B. (2016). Impact of metformin on exercise-induced metabolic adaptations to lower type 2 diabetes risk. *Exercise and Sport Sciences Reviews*.

[42] McArdle A., Pattwell D., Vasilaki A., Griffiths R. D., Jackson M. J. (2001). Contractile activity-induced oxidative stress: cellular origin and adaptive responses. *American Journal of Physiology-Cell Physiology*.

[43] Sampson M. J., Gopaul N., Davies I. R., Hughes D. A., Carrier M. J. (2002). Plasma F_2_ isoprostanes: direct evidence of increased free radical damage during acute hyperglycemia in type 2 diabetes. *Diabetes Care*.

