# Supplementary material for: Timing of Exercise Affects Glycemic Control in Type 2 Diabetes Patients Treated with Metformin
Source: J Diabetes Res. 2018 Mar 29;2018:2483273. doi: 10.1155/2018/2483273 (PMC5896215; doi:10.1155/2018/2483273)
Supplement: Supplementary Materials — Supplementary Table 1: blood glucose concentrations during exercise in EX30, EX60, and EX90. Supplementary Table 2: blood glucose during the four experimental days. [file 2483273.f1.docx]

**Supplementary Table 1** **Blood glucose concentrations during exercise in EX30, EX60 and EX90**

|  | 0 min | 4 min | 8 min | 12 min | 16 min | 20 min | 24 min | 27 min |
| --- | --- | --- | --- | --- | --- | --- | --- | --- |
| EX30 | 8.49 ± 0.32 | 8.56 ± 0.31 | 8.38 ± 0.33 | 7.91 ± 0.37 | 7.80 ± 0.39 | 7.44 ± 0.39 | 5.69 ± 0.43 | 7.25 ± 0.38 |
| EX60 | 9.34 ± 0.42 | 9.10 ± 0.43 | 8.06 ± 0.45* | 7.55 ± 0.43* | 7.00 ± 0.49* | 6.75 ± 0.42* | 6.35 ± 0.39* | 6.07 ± 0.41* |
| EX90 | 8.39 ± 0.51 | 7.78 ± 0.40* | 7.12 ± 0.42* | 6.66 ± 0.43* | 6.14 ± 0.42* | 5.88 ± 0.40* | 5.65 ± 0.38* | 5.48 ± 0.33* |

Values presents as mean ± SEM. *: Compared with EX30, glucose reduction were significantly larger after exercise intervention in EX60 and EX90.

**Supplementary Table 2** **Blood glucose during the four experimental day**

|  | 8:00 a.m | 8:30 a.m. | 9:00 a.m. | 9:30 a.m. | 10:00 a.m. | 12:00 p.m. | 2:00 p.m. | 4:00 p.m. |
| --- | --- | --- | --- | --- | --- | --- | --- | --- |
| Metf | 7.13 ± 0.27 | 8.66 ± 0.36 | 9.92 ± 0.39 | 8.94 ± 0.46 | 7.88 ± 0.33 | 7.23 ± 0.49 | 7.73 ± 0.34 | 7.12 ± 0.32 |
| EX30 | 7.04 ± 0.32 | 8.49 ± 0.32 | 7.25 ± 0.38* | NA | NA | 6.70 ± 0.24 | 7.30 ± 0.24 | 6.84 ± 0.23 |
| EX60 | 6.39 ± 0.22 | NA | 9.34 ± 0.42 | 6.07 ± 0.41* | NA | 6.77 ± 0.26 | 7.30 ± 0.33 | 6.53 ± 0.27 |
| EX90 | 6.60 ± 0.27 | NA | NA | 8.39 ± 0.51 | 5.48 ± 0.33 * | 7.03 ± 0.23 | 7.28 ± 0.32 | 6.77 ± 0.32 |

Values presents as mean ± SEM. *: Compared with Metf, glucose changes were significantly larger after exercise intervention. NA, not available. No significant differences were observed in changes in glucose at 12:00 p.m., 2:00 p.m., and 4:00 p.m.
